# Draft Genome Sequence of the Salt Water Bacterium *Oceanospirillum linum* ATCC 11336^T^

**DOI:** 10.1128/genomeA.00395-17

**Published:** 2017-05-25

**Authors:** Ariel M. Trachtenberg, Joshua G. Carney, Joshua D. Linnane, Bruce A. Rheaume, Natalie L. Pitts, Donald L. Mykles, Kyle S. MacLea

**Affiliations:** a Biology Program, University of New Hampshire, Manchester, New Hampshire, USA; b Department of Biology, Colorado State University, Fort Collins, Colorado, USA; c Biotechnology Program, University of New Hampshire, Manchester, New Hampshire, USA; d Department of Life Sciences, University of New Hampshire, Manchester, New Hampshire, USA

## Abstract

*Oceanospirillum linum* ATCC 11336^T^ is an aerobic, bipolar-tufted gammaproteobacterium first isolated in the Long Island Sound in the 1950s. This announcement offers a genome sequence for *O. linum* ATCC 11336^T^, which has a predicted genome size of 3,782,189 bp (49.13% G+C content) containing 3,540 genes and 3,361 coding sequences.

## GENOME ANNOUNCEMENT

*Oceanospirillum linum* ATCC 11336^T^ is an obligately aerobic, bipolar-tufted gammaproteobacterium taken from the tidal estuary waters of the Long Island Sound in the United States in 1957 by Williams and Rittenberg (1). *O. linum* was combined with *Spirillum atlanticum* (ATCC 12753) and placed in *Oceanospirillum* after the split of the 1832 genus *Spirillum* Ehrenberg, and it was designated the type species for the genus (1–6). The genus currently contains four other named species: *O. multiglobuliferum*, *O. maris*, *O. beijerinckii*, and *O. nioense*. Our research group sequenced *O. linum* (this paper) and *O. multiglobuliferum* (7). Putative *Oceanospirillum* strain MED92 was determined to have <93% *Oceanospirillaceae* sequence similarity at the 16S rRNA gene and was instead assigned to the new genus *Neptuniibacter* as *N. caesariensis* (8).

Unlike freshwater spirilla, *O. linum* is a halophile, capable of growing under conditions of up to 9.75% (wt/vol) (3) or 12% (8) NaCl. It is a strict aerobe, producing polyhydroxybutyrate (PHB) intracellularly, is oxidase and catalase positive, and cannot oxidize or ferment carbohydrates or break down starches, casein, or hippurate (3). *O. linum* is unique in its genus for the ability to use as a sole nitrogen source l-methionine when the cells are provided succinate plus malate as carbon sources (3). Few carbon sources are used (3, 8), although acetate may be used as a sole carbon source when ammonium ions are used as a sole nitrogen source (3).

*O. linum* ATCC 11336^T^ was purchased from ATCC (Manassas, VA, USA) in lyophilized form, rehydrated, and cultured in marine broth or agar (ATCC medium 2216) at 28°C and atmospheric pressure for 48 h. A single colony was grown in log phase, and genomic DNA (gDNA) isolation from these bacteria was achieved using the Genomic-tip 500/G kit (Qiagen, Valencia, CA, USA). The gDNA was fragmented, tagged with adapters using the Nextera DNA library prep kit (Illumina, San Diego, CA, USA), and sequenced with an Illumina HiSeq 2500 sequencer. Two hundred fifty base pair paired-end reads were generated at the Hubbard Center for Genome Studies at the University of New Hampshire (Durham, NH, USA), and Trimmomatic was used for bioinformatic removal of adapter sequences and trimming prior to gene analysis (9).

The genome of *Oceanospirillum linum* was assembled from 12,571,740 reads into 289 contigs using SPAdes version 3.8.0 (10). These contigs were interpreted with QUAST version 4.1 to have a total length of 3,782,189 bp, a G+C% of 49.13%, and an average coverage of 1,702× (11). The largest contig found was 1,127,340 bp, with an *N*_50_ value of 573,653 bp. The G+C% results are in strong agreement with previous reports of G+C content of 48% (3, 12, 13) and 49% (14).

The National Center for Biotechnology Information (NCBI) automatic annotation pipeline (PGAP) was used for genome annotation (15). A total of 3,540 genes, 3,361 coding sequences (CDSs), 96 RNA genes, 83 pseudogenes, and 3 clustered regularly interspaced short palindromic repeat (CRISPR) arrays were discovered by PGAP.

### Accession number(s).

This whole-genome shotgun project has been deposited at DDBJ/EMBL/GenBank under the accession no. MTSD00000000. The version described in this paper is version MTSD02000000.
